# The oncoprotein and stem cell renewal factor BMI1 associates with poor clinical outcome in oesophageal cancer patients undergoing preoperative chemoradiotherapy

**DOI:** 10.1186/1471-2407-12-461

**Published:** 2012-10-09

**Authors:** Reigetsu Yoshikawa, Tohru Tsujimura, Lihua Tao, Norihiko Kamikonya, Yoshinori Fujiwara

**Affiliations:** 1 Department of Surgery, Kanzaki Hospital, 3-1-10, Hama, Amagasaki, Hyogo, 661-0967, Japan; 2 Department of Pathology, Hyogo College of Medicine, 1–1, Mukogawa-cho, Nishinomiya, Hyogo, 663-8501, Japan; 3 Department of Radiology, Hyogo College of Medicine, 1–1, Mukogawa-cho, Nishinomiya, Hyogo, 663-8501, Japan; 4 Department of Digestive Surgery, Nara Hospital, Kinki University school of Medicine, 1248-1, Otoda-cho, Ikoma, Nara, 630-0293, Japan

**Keywords:** Cancer stem cell, Chemoradiotherapy (CRT), BMI1, Hedgehog (Hh), Oesophageal cancer, p16^INK4A^

## Abstract

**Background:**

The polycomb group (PcG) family BMI1, acting downstream of the hedgehog (Hh) pathway, plays an essential role in the self-renewal of haematopoietic, neural, and intestinal stem cells, and is dysregulated in many types of cancer. Our recent report has demonstrated that Hh signalling activation can predict very earlier relapse of oesophageal cancers. As data were not available on the clinical role of BMI1 expression in oesophageal cancers after chemoradiotherapy (CRT), we analysed whether it could be also used to predict disease progression and prognosis in oesophageal cancer patients undergoing trimodality therapy of preoperative CRT and oesophagectomy.

**Methods:**

Expressions of BMI1 and p16^INK4A^, a downstream target of PcG, were analysed in 78 patients with histologically confirmed oesophageal squamous cell carcinoma (ESCC) after preoperative CRT by immunohistochemical staining. The association of BMI1 and p16^INK4A^ expression with clinicopathologic characteristics was analysed by *χ*^2^-test. Survival analysis was carried out by the log-rank test using Kaplan-Meier method.

**Results:**

Among 78 ESCC patients, 24 patients (30.8%) showed BMI1 positivity, mainly localised in the nuclei of tumour cells. Patients harbouring BMI1-positive tumour cells showed significantly poorer prognoses than those without such cells or residual tumours (mean disease-free survival (DFS) time 16.8 *vs* 71.2 months; 3-yr DFS 13.3% *vs* 49.9%, *P*=0.002; mean OS time 21.8 *vs* 76.6 months; 3-yr OS 16.2% *vs* 54.9%, *P*=0.0005). There was no significant correlation between p16^INK4A^ expression and BMI1 expression.

**Conclusions:**

Our study shows that BMI1 expression is a predictor of early relapse and poor prognosis in ESCC after CRT. These findings suggest that BMI1 signal activation might be involved in promoting cancer regrowth and progression after CRT, and might be indicative of emergence of ‘more aggressive’ cancer progenitor cells.

## Background

Oesophageal cancer is a virulent malignancy that has a high fatality rate once it progresses beyond disease Stage I. The presence of lymph node metastasis and vascular invasion as well as distant organ involvement leads to a poor clinical outcome [1]. The optimal management of oesophageal cancer is complicated because both global and institutional standard therapies vary and patients often need specific individual management strategies. There are data to support multimodal approaches for this disease. Surgery is considered the mainstay of treatment for patients, especially those with locoregionally confined oesophageal cancer. However, the 5-year survival rate is generally 20–50%, mainly due to the high rate of recurrence, even after curative surgery [1,2]. In Japan, preoperative chemotherapy has been proven to be associated with a better clinical outcome than postoperative chemotherapy (randomised trial of the Japan Esophageal Oncology Group, JCOG9907) [3]. Moreover, preoperative chemoradiotherapy (CRT) has been accepted as a first-line therapy in the Unites States [4]. Since 1996, we have advocated a trimodal therapy, a combination of radical surgery with preoperative CRT, and have reported increased resectability, reduced incidence of both local recurrence and distant metastasis, and better clinical outcome for CRT responders [5]. However, the benefit of preoperative CRT over chemotherapy alone has not yet been established in Japan, and remains controversial in Europe. Some clinical trials in the West have shown that this preoperative strategy benefits only the 25% of patients who show a pathological complete response (CR; no cancer cells in the resected specimen), whereas the remaining 75% present with CRT-resistant and highly aggressive cancers with lymph node and distant metastases [6,7]. High mortality from this disease after surgery is due to the limited number of current therapies, and refractoriness of the disease due to the emergence of therapy-resistant cancer cells. Therefore, it is clearly imperative to develop biomarkers for predicting recurrence and to implement efficient treatments for preventing recurrence after surgery.

Therapy-resistant cancer cells are thought to be the founder population that causes patient relapse and subsequent metastasis. A recent ‘cancer stem cell’ concept has suggested the existence of a small subpopulation of tumour cells harbouring stem cell-like unique properties of self-renewal, asymmetric division, and pluripotency. It is thought that these ‘more aggressive’ cancer progenitor cells, refractory to conventional therapy, survive the treatment to later regenerate a tumour with increased resistance to therapy [8]. To date, putative cancer stem cells have been identified in various cancer types including breast [9], lung [10], colon [11,12], and pancreatic cancer [13]. To treat the patients who harbour these cancer stem cells, chemo(radio)therapy and/or molecular-targeted therapy, which has power to kill these cells, is essential.

B-cell-specific Moloney murine leukaemia virus integration site 1 (*Bmi1*) was first identified by retroviral insertional mutagenesis when assessing collaborating oncogenes in Eμ-myc transgenic mice [14,15]. Bmi1 is a transcriptional repressor that belongs to the polycomb group (PcG) family of proteins involved in axial patterning, haematopoiesis, regulation of proliferation, and senescence [16]. It has been shown to play a role in sustaining self-renewing cell activity by repressing the INK4A locus encoding *p16*^*INK4A*^ and *p19*^*ARF*^, which are capable of inducing growth arrest, cellular senescence and apoptosis [17-19]. PcG proteins are known to modify the chromatin structure around their binding sites by marking the chromatin of their target genes through methylation at lysine 27 of histone H3; these sites include the promoters of many developmental regulator genes, leading to gene repression [20]. Recent studies have documented increased *BMI1* expression in a variety of human cancers, such as non-small cell lung cancers [21], medulloblastomas [22], prostate carcinomas [23], colorectal cancers [24], breast carcinomas [25], and oesophageal squamous cell carcinomas (ESCCs) [26]. Furthermore, BMI1, as well as Gli-1 of the hedgehog (Hh) pathway, has been shown to be a key regulator of self-renewal in both normal and tumourigenic human mammary stem cells [27]. In our recent study, we have shown the clinical significance of Hh signal activation to predict very earlier relapse and poorer prognosis in patients with ESCC after CRT [28]. Hence, aberrant BMI1 expression might also be involved in the characteristics of the ‘more aggressive’ cancer cell population after CRT, because BMI1 is thought to be a downstream target in the Hh pathway in medulloblastoma [22].

No data are currently available on the role of BMI1, a candidate downstream target of the Hh pathway, in oesophageal cancer progression after CRT. In this study, therefore, we retrospectively investigated the expression of BMI1 protein in human oesophageal cancer tissues and evaluated the clinical implications of aberrant BMI1 activation for these patients who underwent preoperative CRT and oesophagectomy.

## Methods

### Patients and therapy

Between April 1996 and December 2005, 78 patients, 13 women and 65 men with a mean age of 62.0 years (range, 38–78 years), with surgically excised oesophageal cancer were studied at the Hyogo College of Medicine, Japan. For preoperative CRT, chemotherapy consisted of 5-flurouracil (5-FU; 500 mg/m^2^ per day) administration for a 120-h continuous intravenous (i.v.) infusion starting on day 1, and cisplatin (CDDP; 15 mg/m^2^ per day) for a 2-h i.v. infusion on days 1–5, as described previously [28,29]. Concurrent radiation therapy was performed after CDDP infusion on days 1–5 by using a linear accelerator (Mevatron KD2, Siemens, Germany), as described previously [5]. Chemotherapy was combined with radiation therapy during the first week, and then radiation therapy alone was repeated for the next 3 weeks (days 8–12, 15–19, and 22–26). The patients received 20 fractions of 2 Gy/day for a total dose of 40 Gy. Surgery was usually performed 4–6 weeks after the completion of CRT. After the surgery, monthly follow-up at the outpatient clinic was scheduled. Other relevant patient information was obtained from office charts, hospital records, and telephone interviews. Prior to the use of these clinical materials for investigation, approval from the institutional ethics committee (Hyogo College of Medicine) and informed consent from patients were obtained.

### Evaluation prior to surgery

Approximately 3–5 weeks after the completion of CRT, patients underwent a complete staging workup. Patients were defined to have clinical CR to CRT if no residual tumour was detected by endoscopy and if no occurrence of metastatic disease was identified on a computed tomography (CT) scan evaluation.

### Immunohistochemistry

ESCC tissue specimens obtained by surgical resection after preoperative CRT were cut longitudinally, and fixed in 10% formalin-solution. The pieces of ESCC tissue were processed using conventional procedures for paraffin embedding, and cut into 5-μm thickness. Specimens were heated for 20 min at 98°C in Target Retrieval Solution pH 9 (S2368, DakoCytomation, Glostrup, Denmark) to facilitate antigen retrieval. They were then incubated with mouse monoclonal antibody against human BMI1 (F6, Upstate, Lake Placid, NY, USA, diluted 1:100 in Dako REAL Antibody Diluent [S202230, Dako, Glostrup, Denmark]), mouse monoclonal antibody against human p16^INK4A^ (F-12, Santa Cruz Biotechnology, Santa Cruz, CA, USA, 1:250), and goat polyclonal antibody against human Gli-1 (C-18, Santa Cruz Biotechnology, 1:500), and sequentially with an anti-mouse immunoglobulin antibody using ChemMate EnVision Kit (DakoCytomation). Immunoreacted cells were visualized with 3, 3’-diaminobenzidine, and nuclei were lightly counterstained with haematoxylin. Normal mouse immunoglobulin G (IgG) was substituted for the primary antibody as a negative control. Sections were examined microscopically by two pathologists of the authors (L.T. and T.T.) without prior knowledge of clinicopathological features. Immunohistochemical samples were graded by the presence of positively stained tumour cells as follows: no specific staining or <5% tumour cells (−); ≥5% to <35% tumour cells (+); ≥35% to <65% tumour cells (++); and ≥65% tumour cells (+++).

### Statistical analysis

All statistical analyses were carried out using STATISTICA statistical software, version 06 J (STATISTICA, Tulsa, OK, USA). Overall survival (OS) was defined as the time from initial diagnosis to either the patient’s death or the date of the last available information on vital status. Disease-free survival (DFS) was defined as the length of time after treatment during which no cancer was found. In univariate analysis, the difference between the cumulative survival rates of the patient groups was calculated by the log-rank test for comparison using Kaplan-Meier survival curves. *P* < 0.05 was considered statistically significant.

Retrospective studies generally utilise the same archived materials several times. This study is about the ongoing therapeutic strategy, and patient characteristics were not the same as that described in the previous study published in 2008 [28]. The present study includes data of an additional 9 patients subjected to CRT after the previous evaluation, leading to the updated univariate analysis of prognostic factors for survivals. Supplementary Gli-1 data used in contrast with BMI1 has been re-evaluated for this study. Evaluation for each clinical study was dealt with independently.

## Results

### Patient and tumour characteristics

The patient characteristics are summarised in Table 1. The patient gender bias was male dominated (male: female = 65:13). The mean follow-up period was 30 months (range, 3–120 months). One patient was lost to follow-up. The tumour histology was ESCC in all cases, with 75 tumours (96.2%) originating in the thorax. On the basis of the tumour-node-metastasis (TNM) system of the International Union Against Cancer (UICC), stage II tumours were seen in 30 patients (38.5%), stage III tumours were seen in 32 patients (41.0%), and stage IV tumours were seen in 16 patients (20.5%). In total, 34 patients (43.6%) had lymph node metastasis at the time of diagnosis. All lesions before CRT presented with a T_3_ or T_4_ extent of invasion. Three-quarters of the patients had tumours that were between 6 and 8 cm in diameter. An M+ classification was described in 12 tumours. Three patients had distant metastasis of the liver. All patients experienced a disease-free period. During the follow-up period, 17 patients (21.8%) developed local recurrence or residual tumours, ten patients (12.8%) developed neck or celiac lymph-node recurrence, and 14 patients (17.9%) developed distant metastasis. In total, 41 patients (52.6%) died during follow-up; among these, 33 patients (42.3%) died from their tumours, while the remaining eight patients (10.3%) were tumour free and died of intercurrent diseases.

**Table 1:** Patient characteristics

**Characteristics**		***n***
Sex (Male/Female)		78 (65/13)
Mean age, years (range)		62.0 (38–76)
Location of tumour	Cervix	3
	Upper thorax	10
	Middle thorax	44
	Lower thorax	21
T-classification	T_3_	40
	T_4_	38
N-classification	N_0_	44
	N_1_	34
M-classification	M_0_	66
	M_1_	12
UICC TNM stage	IIa	30
	III	32
	IVa	8
	IVb	8

UICC: International Union against Cancer.

### BMI1 positivity predicts early recurrence and poor prognosis

**Figure 1:**
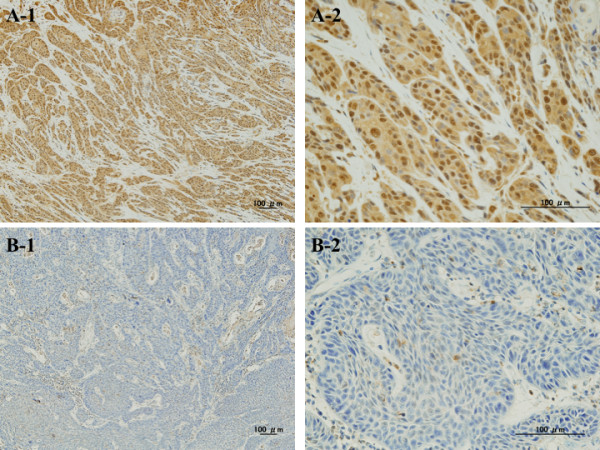
**Immunohistochemical detection of BMI1 in ESCC specimens.A**. Strong expression of BMI1 (A-1; ×10, A-2; ×40). **B**. Null expression of BMI1 (B-1; ×10, B-2; ×40). Bars indicate 100 μm.

Twenty-eight tumours were completely eradicated by CRT, resulting in an absence of visible tumour cells: a pathological CR. BMI1 immunostaining was diffuse and granular, and predominantly localised in the nuclei of ESCCs. BMI1 expression was absent from 26 tumours and 28 eradicated regions but present in 24 tumours (30.8%); 14 tumours (17.9%) were for grade (+), 8 (10.3%) for (++), and 2 (2.6%) for (+++) (Figure 1). All eight cancers with Gli-1-positive nuclei expressed BMI1. In the BMI1-positive group, recurrences were found in 18 out of 24 patients (75.0%); these were local in seven cases and distant (in the liver, bone, lung and neck lymph nodes) in 11 cases. By contrast, in the BMI1-negative or no residual tumour group (*n* = 54), recurrences were found in 24 patients (in the liver, bone, skin, neck lymph nodes and thyroid gland); these were local in 16 cases and distant in eight cases. Statistical analysis by χ^2^-test showed that BMI1 positivity significantly correlates with T and N staging (*P* = 0.0137, and *P* = 0.0130, respectively) but not with M staging (*P* = 0.755). According to the subanalysis, distant recurrence was more common in patients positive for BMI1 (*P* = 0.0084). The univariate analysis of the OS prognostic factors is summarised in Table 2. Lymph node metastasis, distant metastasis and depth of tumour invasion all had significant prognostic value (*P* = 0.002, *P* = 0.0001, and *P* = 0.0001, respectively). Furthermore, patients showing an effect of CRT revealed significantly better OS prognosis compared with those showing no effect (*P* = 0.0001).

**Table 2:** Univariate analysis of prognostic factors for OS

**Covariate**		***n***	**Hazard ratio**	**95%CI**	***P-*value for OS**
Age (years)	≥70	22	1.111	0.552-2.234	0.770
	<70	56			
Gender	Male	65	0.312	0.096-1.016	0.023*
	Female	13			
Effect of CRT	Effective	56	0.191	0.098-0.370	0.0001***
	Not effective	22			
Lymph-node	Positive	34	2.624	1.382-4.983	0.002**
metastasis	Negative	44			
Distant metastasis	Positive	12	3.988	2.001-7.947	0.0001***
	Negative	66			
Depth of tumour	T_3_	40	0.308	0.157-0.602	0.0001***
invasion	T_4_	38			
Tumour location^1^	Upper	13	1.299	0.597-2.827	0.509
	Lower	65			
BMI1 expression	positive	24	3.347	1.775-6.313	0.0001***
	negative	54			

^1^ Upper or lower: above or below the tracheal bifurcation.

*P<0.05; ** *P*<0.005; *** *P*<0.0005.

Kaplan-Meier analysis suggested that the prognosis was particularly unfavourable for patients with BMI1 positivity in their primary tumours (Group III), compared with BMI1-negative tumour patients (Group II) or those with no residual tumour (pathological CR; Group I).

**Figure 2:**
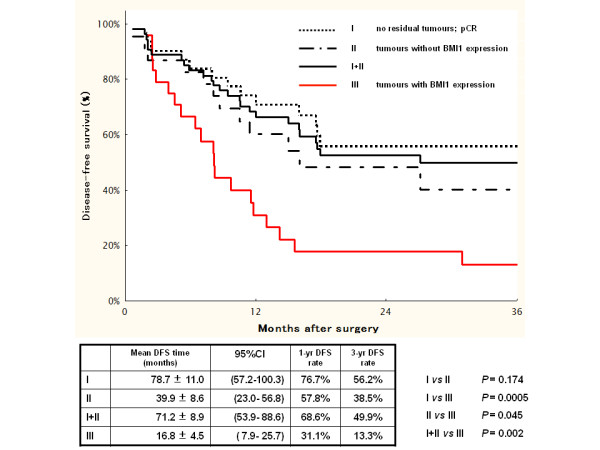
**DFS curves and *P*-values (log-rank test) for comparisons among patients with no residual tumour (Group I), those with BMI1-negative tumours (Group II), patients with no residual tumour and those with BMI1-negative tumours combined (Group I+II), and patients with BMI1-positive tumours (Group III).**

**Figure 3:**
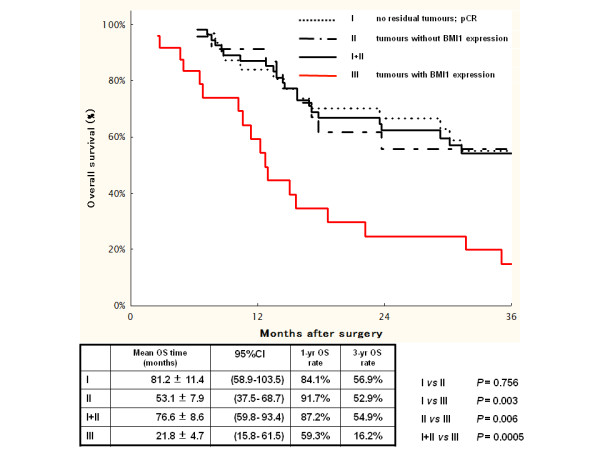
**OS curves and *P*-values (log-rank test) for comparisons among patients with no residual tumour (Group I), those with BMI1-negative tumours (Group II), patients with no residual tumour and those with BMI1-negative tumours combined (Group I+II), and patients with BMI1-positive tumours (Group III).**

The mean DFS time in the BMI1-positive tumour group (Group III) was 16.8 ± 4.5 months (95% CI 7.9–25.7 months) compared with 71.2 ± 8.9 months (95% CI 53.9–88.6 months) in the BMI1-negative tumour group and the no residual tumour group combined (Group I+II) (Figure 2; *P* = 0.002); the mean OS times for these groups were 21.8 ± 4.7 months (95% CI 15.8–61.5 months) (Group III) and 76.6 ± 8.6 months (95% CI 59.8–93.4 months) (Group I+II), respectively (Figure 3; *P* = 0.0005). Furthermore, the BMI1-positive tumour group (Group III) showed significantly shorter DFS and OS times compared with the BMI1-negative tumour group alone (Group II: Figure 2; mean DFS time 39.9 ± 8.6 months, 95% CI 23.0–56.8 months, *P* = 0.045, Figure 3; mean OS times 53.1 ± 7.9 months, 95% CI 37.5–68.7 months, *P* = 0.006).

The 1-year and 3-year DFS rates in the BMI1-positive group (Group III) were 31.1% and 13.3% respectively. Those in the BMI1-negative tumour group (Group II) were 57.8% and 38.5%, while those in the BMI1-negative and no residual tumour groups combined (Group I+II) were 68.6% and 49.9%, respectively. The corresponding 1-year and 3-year OS rates were 59.3% and 16%, respectively, in Group III, 91.7% and 52.9%, respectively, in Group II, and 87.2% and 54.9%, respectively, in Group I+II.

### Correlation between BMI1 and p16^INK4A^

The protein p16^INK4A^ was considered to be present when nuclear and/or cytoplasmic staining was detectable. Of 78 ESCC patients subjected to CRT, 55.2% were completely negative (category 0), 25.6% were weakly positive (category I, 5-50% positive cells) and 19.2% were strongly positive (category II, 51-100% positive cells). Statistical analysis by χ^2^-test showed that p16^INK4A^ positivity was not correlated with BMI1 positivity (*P* = 0.7662). Furthermore, there was no correlation between p16^INK4A^ positivity and prognosis in BMI1-positive tumours (*P* = 0.4602).

## Discussion

We have shown that persistent expression of the chemokine receptor CXCR4 and Hh signal activation can predict very earlier relapse and poorer prognosis in ESCC patients after CRT [28,30]. Our findings suggest that stem cell renewal factors are, at least in part, but critically, involved in recurrent and metastatic behaviour under the microenvironment (niche) around the surfaces of tumour cells, in response to external therapeutic intervention (for example, CRT). Additionally, the current study has revealed that expression of BMI1 protein along with Hh signal activation might be a useful and reliable biomarker for the screening and management of high-risk patients with poorer prognosis after CRT. In the patients with BMI1-negative tumours, postoperative chemotherapy seemed to prolong the survival time to the same level achieved in the no residual tumour group, even after the diagnosis of a recurrence. Glinsky *et al*. demonstrated that tumours displaying a stem cell-like expression profile, namely, an 11 *BMI1* oncogene-driven gene signature, have a significantly higher probability of developing distant metastasis within five years after therapy in five epithelial malignancies (prostate, breast, lung, ovarian, and bladder cancers) and five non-epithelial malignancies such as lymphoma [23], and that the CD44+/CD24- blood-borne human prostate carcinoma metastasis precursor cell population contains a large proportion of cancer cells with high-level coamplification of both *BMI1* and *Ezh2* PcG genes [31]. A growing body of preclinical and clinical evidence is accumulating to show the significance of BMI1 in chemoresistance and recurrence [32]. Taken together, these data strongly support the possibility of a causal association between Hh-PcG pathway activation and manifestation of various clinically lethal, therapy-resistant cancer phenotypes. Intriguingly, 70.8% of BMI1-positive tumours in the present study also expressed CD44 strongly (data not shown). In the terminal stage, the network of stem cell surface markers implicating cancer progenitor cells might epigenetically contribute to cancer relapse and treatment failure.

To date, there has been no specific decision-making strategy in clinical management for oesophageal cancer patients after CRT. Immunohistochemical analysis of BMI1 and/or Gli-1 in all post-CRT ESCC patients seems highly informative and convenient in the stratification of patients into subgroups with a distinct likelihood of therapy failure. In some cases, supportive care emphasising quality of life rather than aggressive anti-cancer therapy might be recommended, or a pioneering clinical trial utilising Hh-PcG chromatin-silencing pathways blockade should be prioritised in high-risk patients. Despite the recent advances in oesophageal cancer treatment, the fact remains that clinicians continue to face the challenge of frequent recurrence or metastasis relating to CRT-resistant ‘more aggressive’ cancer cells after surgery. Namely, it is suggested that the limitation of present therapies is likely to relate to their inability to target the cancer stem cell component, and that now is the time for a paradigm shift in anti-cancer strategy. Sims-Mourtada *et al*. demonstrated *in vitro* that Hh-induced chemoresistance through multiple drug transporter mechanisms can be overcome by inhibitors of the Hh pathway [33]. Furthermore, Berezovska *et al*. showed potential therapeutic efficacy of the siRNA-mediated targeting of BMI1 expression, *in vitro* and *in vivo*, for treating metastatic prostate cancer [34]. When the molecular mediators of therapeutic resistance by cancer stem cells are established, developing clinically efficient and safe inhibitors to target these pathways, focusing on Hh-PcG pathway activation, would definitely be of benefit for the treatment of high-risk patients.

In this study, we also found no correlation between BMI1 positivity and p16^INK4A^ positivity. In contrast to the results of our study, BMI1-mediated regression of *p16*^*INK4A*^ has been reported to contribute to an increased incidence of metastasis in melanoma patients [35]. Furthermore, the loss of *p16*^*INK4A*^ gene expression, mainly caused by frequent *p16*^*INK4A*^ promoter methylation, is known to have a strong relationship with poor prognosis in ESCC patients [36]. The contradictory result of no direct linking between BMI1 and downstream p16^INK4A^ in this study may be mainly due to the effects of therapeutic intervention. CRT can be assumed to cause G1-S phase cell cycle alteration which could interfere in the regression of p16^INK4A^, involving ‘more aggressive’ cancer cells *per se* and their indispensable microenvironment niche. Thus, in ESCC patients subjected to CRT, the role of BMI1-mediated inhibition of p16^INK4A^ protein is not clear, and the clinical effect of Hh signalling pathway activation might be more evident than that of the presence of BMI1, whilst BMI1and Gli-1 both play important roles in cancer progression.

Increased understanding of the importance of stem cell-related markers allows a more targeted approach to therapy for individual tumours. Our current study highlights the novel therapeutic targeting of distinct oncogenic signalling elements activated in cancer progenitor cells and their local microenvironment. In this paper, the direct effect of CRT for BMI1 expression was not investigated. Liu *et al*. have shown that BMI1 expression was observed in 64.3% of ESCC patients [26], whereas after CRT, expression was observed in 30.8% of the patients in our study. These results suggested that persistent BMI1 expression even after CRT correlated with earlier relapse and treatment resistance, and that CRT was contributory in eradicating ‘more aggressive’ cancer cells aberrantly overexpressing BMI1. This is of great importance to propose a practical implementation of the concept of (semi-) personalised treatment in the clinical field of action.

## Conclusions

We demonstrated that BMI1 positivity indicative of PcG protein chromatin silencing pathway activation was significantly correlated with oesophageal cancer recurrence and poor prognosis after CRT, and that it was not inversely correlated with the presence of the downstream target p16^INK4A^. Although the functional and clinical significance of the stemness signature, including components of the Hh and PcG pathways, remain to be elucidated, we suggest that BMI1 expression along with Gli-1-positive expression is a link between relapse and CRT-resistant cancer cells, and is thus a potential prognostic biomarker and rational therapeutic target for attacking the ‘more aggressive’ cancer cells causing recurrence and treatment resistance.

## Abbreviations

CRT: Chemoradiotherapy; ESCC: Oesophageal squamous cell carcinoma; 5-FU: 5-fluorouracil; CDDP: Cisplatin.

## Competing interests

The authors declare that they have no competing interests.

## Authors’ contributions

RY and YF were responsible for experimental design, data collection and analysis, interpretation of the results, and writing the manuscript. TT and LT were responsible for reviewing and scoring the degree of immunohistochemistry. NK conducted radiotherapy, and participated in experimental design and interpretation. All authors have read and approved the final manuscript.

## Authors’ information

RY and YF were formerly with Department of Surgery, Hyogo College of Medicine.

## Pre-publication history

The pre-publication history for this paper can be accessed here:

http://www.biomedcentral.com/1471-2407/12/461/prepub
